# Visual-motor integration in children with unilateral cerebral palsy: application of the computer-aided measure of visual-motor integration

**DOI:** 10.1186/s12984-024-01335-8

**Published:** 2024-03-19

**Authors:** Wen-Feng Huang, Ren-Yu Chen, Tien-Ni Wang, Po-Ya Chuang, Jeng-Yi Shieh, Hao-Ling Chen

**Affiliations:** 1 School of Occupational Therapy, College of Medicine, National Taiwan University, No. 17, Xu-Zhou Rd. 4 Floor, Taipei City, 100 Taiwan, ROC; 2 Center of Child Development, Far Eastern Memorial Hospital, New Taipei City, Taiwan; 3 Department of Physical Medicine and Rehabilitation, National Taiwan University Hospital, Taipei City, Taiwan; 4 Department of Rehabilitation, Show Chwan Memorial Hospital, Changhua, Taiwan

**Keywords:** Unilateral cerebral palsy, Children, Visual-motor integration, Beery-VMI, Handwriting, Writing readiness, Computer-aided, Technology, Image registration

## Abstract

**Background:**

Children with unilateral cerebral palsy (UCP) are encouraged to participate in the regular school curriculum. However, even when using the less-affected hand for handwriting, children with UCP still experience handwriting difficulties. Visual-motor integration (VMI) is a predictor of handwriting quality. Investigating VMI in children with UCP is important but still lacking. Conventional paper-based VMI assessments is subjective and use all-or-nothing scoring procedures, which may compromise the fidelity of VMI assessments. Moreover, identifying important shapes that are predictive of VMI performance might benefit clinical decision-making because different geometric shapes represent different developmental stepping stones of VMI. Therefore, a new computer-aided measure of VMI (the CAM-VMI) was developed to investigate VMI performance in children with UCP and to identify shapes important for predicting their VMI performance.

**Methods:**

Twenty-eight children with UCP and 28 typically-developing (TD) children were recruited. All participants were instructed to complete the CAM-VMI and Beery-Buktenica Developmental Test of Visual-Motor Integration (Beery-VMI). The test items of the CAM-VMI consisted of nine simple geometric shapes related to writing readiness. Two scores of the CAM-VMI, namely, Error and Effort, were obtained by image registration technique. The performances on the Beery-VMI and the CAM-VMI of children with UCP and TD children were compared by independent *t*-test. A series of stepwise regression analyses were used to identify shapes important for predicting VMI performance in children with UCP.

**Results:**

Significant group differences were found in both the CAM-VMI and the Beery-VMI results. Furthermore, *Error* was identified as a significant aspect for predicting VMI performance in children with UCP. Specifically, the square item was the only significant predictor of VMI performance in children with UCP.

**Conclusions:**

This study was a large-scale study that provided direct evidence of impaired VMI in school-aged children with UCP. Even when using the less-affected hand, children with UCP could not copy the geometric shapes as well as TD children did. The copied products of children with UCP demonstrated poor constructional accuracy and inappropriate alignment. Furthermore, the predictive model suggested that the constructional accuracy of a copied square is an important predictor of VMI performance in children with UCP.

## Background

Children with unilateral cerebral palsy (UCP), which causes non-progressive impairments of the unilateral body, are expected to integrate into mainstream schools and participate in the regular classroom curriculum [1, 2]. Children with UCP mainly use their less-affected hands to complete most daily activities at school, including handwriting, drawing, and other handwriting-related tasks [2]. For school-aged children, a significant part of their school time is allocated to handwriting-related tasks [3]. Even when using the less-affected hand for handwriting, however, children with UCP still experience difficulties in handwriting quality [1, 2, 4]. Visual-motor integration (VMI), the ability to perceive stimuli from visual input and appropriately integrate the information to the motor response, is reported to be a predictor of handwriting quality [5]. Investigation of VMI in children with UCP may increase understanding of the mechanism of handwriting difficulties in children with UCP [1].

Despite the importance of VMI, few studies have investigated VMI performance in children with UCP [6]. These studies mainly focused on investigating visual-motor skills during the reach-to-grasp task. The results revealed that children with UCP had difficulties in accurately reaching to grasp a target while using the less-affected hand with appropriate gaze patterns [7, 8]. However, the VMI involved in such a gross-motor task (e.g., reach-to-grasp) might be different from that in fine-motor tasks (e.g., handwriting, drawing, and other handwriting-related tasks). Some studies have proposed VMI interventions for children with UCP, periphrastically implying VMI deficits in these children [6, 9, 10]. Although the impaired VMI in children with UCP has been taken for granted, direct evidence should be provided. Only one pilot study has directly investigated VMI performance in children with UCP, but the preliminary findings were compromised due to the small sample size (*N* ≤ 4) and limited strength of the evidence [11]. A large-scale study comparing VMI performance between children with UCP and typically developing (TD) children is needed.

VMI performance is typically evaluated with paper-based assessments such as the Beery-Buktenica Developmental Test of Visual-Motor Integration (Beery-VMI) [12]. In these assessments, several geometric shapes of varying difficulty are copied and scored based on developmental criteria and group samples. The fidelity of these assessments is usually compromised due to the use of subjective hand-scoring and crude visual inspection. Moreover, the widely-used all-or-nothing scoring procedure may not differentiate subtle differences in VMI ability between individuals having the same scores; thus, the sensitivity of scoring is limited. The hand-scoring procedure is also time-consuming and unavoidably entails human error [13]. To overcome the above limitations, computer-aided techniques have become available for VMI evaluation [13–15]. Recently, our team developed a computer-aided VMI assessment for children with UCP. It is hoped that this assessment can offer valuable insights for VMI evaluation and serve as a supplement to paper-based VMI assessments.

Apart from the scoring system, the shapes to be copied, which could influence VMI performance, should also be considered. Previous factor analytic studies suggested that different structural characteristics of the test items should be noted in the VMI evaluation [16, 17]. Closed shapes with acute and oblique angles might represent different contributions to VMI performance than would items consisting of simple horizontal and vertical lines. On the other hand, from the perspective of development, copying different shapes requires different visual-motor skills and represents different developmental stepping stones [12, 18]. For example, copying a circle might require directional awareness, and copying a cross might require the ability to cross the midline of the body [12]. Considering the implicit attributes of VMI in relation to the structural characteristics of different shapes, investigation of the contributions of different shapes to VMI performance would be helpful for evaluating VMI performance in children with UCP.

The purposes of this study were organized into two tiers. In the first tier, the VMI performances of school-aged children with UCP and TD children were explored by computer-aided VMI assessment and Beery-VMI. In the second tier, the shapes important for predicting VMI performance in children with UCP were identified.

## Methods

### Participants

A convenience sample of 56 children, including 28 children with UCP and 28 TD-controlled children, were recruited. Children with UCP were recruited according to the following criteria: (1) a clinical diagnosis of UCP, (2) school placement within a typical class for ages 6 to 12 years, (3) no excessive muscle tone at any joint of the upper limb (Modified Ashworth Scale ≤ 2), and (4) ability to follow instructions and complete the VMI tasks. In addition, age- and gender-matched TD children, without a history of any developmental disorders, were also recruited. This study was approved by the Research Ethics Committee of the National Taiwan University Hospital. All children and caregivers provided written informed consent before participating in the study.

### Measuring instruments

Two assessment tools, namely, the Computer-Aided Measure of Visual-Motor Integration (CAM-VMI) and the Beery-VMI (fourth edition), were used to investigate VMI performance.

#### Beery-Buktenica developmental test of visual-motor integration test, fourth edition

The Beery-VMI is a standardized tool that is widely used to evaluate VMI performance in school-aged children [6, 12, 19]. This test is paper-based and consists of 24 items to be copied. The test items are geometric shapes arranged in a developmental sequence. In this study, the participants were instructed to copy these items into the corresponding grid on the sheet to the best of their ability until 3 consecutive products did not receive passing scores. In the binary scoring (i.e., pass or fail), one point was awarded for the correct product and zero points were given for failure [12]. The raw scores of the Beery-VMI were calculated as the number of correctly copied products (score range 4–27), with higher scores indicating better VMI performance.

##### Computer-aided measure of visual-motor integration (CAM-VMI)

#### Item selection

Nine geometric shapes that were reported as indicators of writing readiness were selected as the test items of the CAM-VMI [18, 20]. The items included lines (horizontal, vertical, left oblique, and right oblique lines), cross (cross and oblique cross), and closed shapes (circle, square, and triangle). Participants were instructed to copy the nine items as accurately as possible.

#### Scoring system

**Fig. 1:**
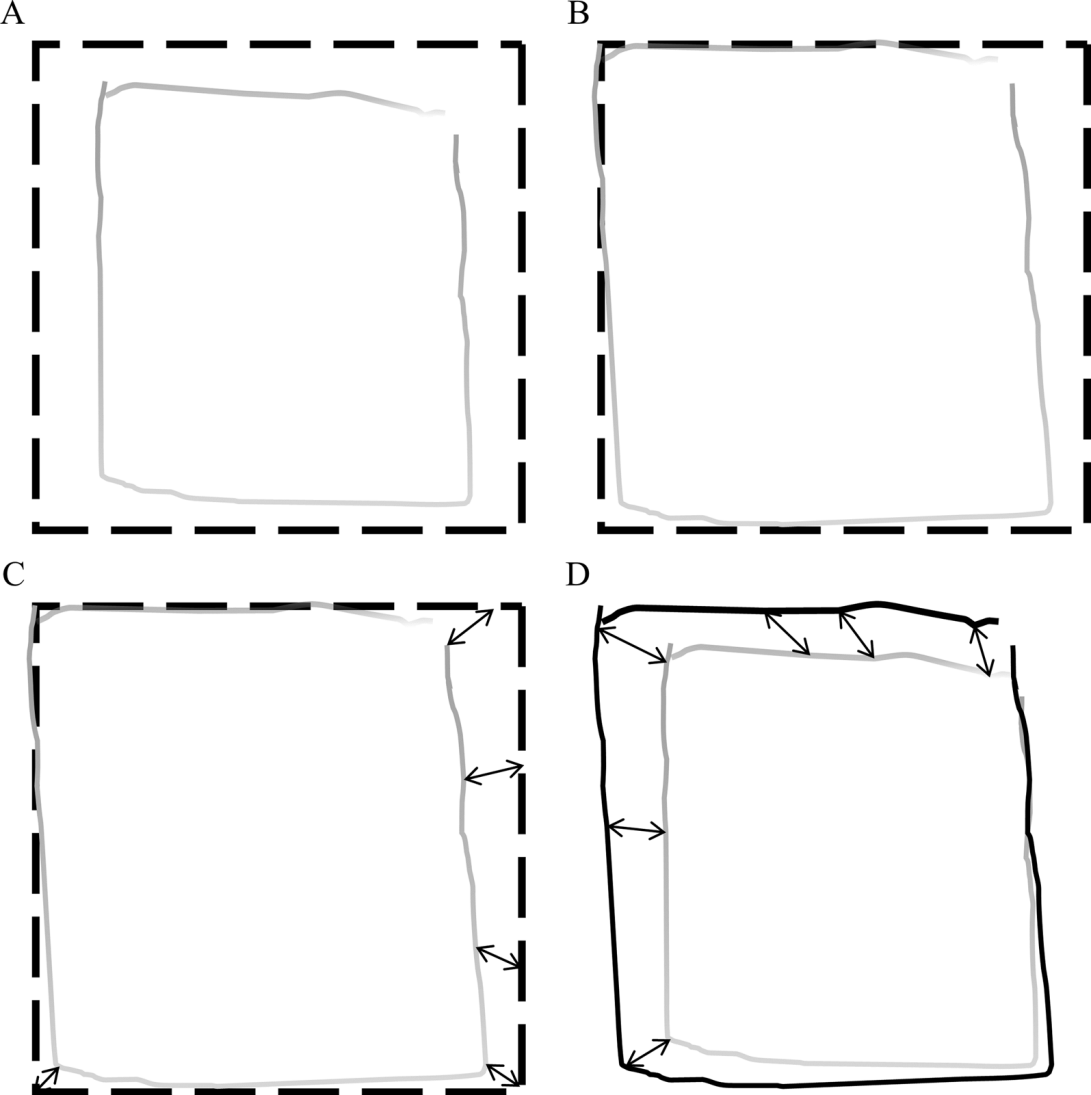
Flow chart of image registration technique in the CAM-VMI. **A** The copied product (solid-gray pattern) superimposed onto the template (dashed-black *square*), **B** The two images superimposed after optimal resizing, rotation, and repositioning, **C** *Error* reflects the constructional accuracy by comparing the template (dashed-black *square*) and the best-fit (solid-black pattern), and **D** *Effort* reflects the required adjustment by comparing the best-fit (solid-black pattern) and the original copied product (solid-gray pattern)

To objectively quantify the VMI performance, the scoring system of the CAM-VMI was developed based on the image registration technique. The image registration technique has been adopted in the evaluation of Chinese handwriting legibility and demonstrated its advantages in clinical usage [21]. Each copied product was scanned as a digital image and optimally registered to the corresponding template. An optimization routine was adopted in the CAM-VMI to find the best-fit alignment that yielded optimal similarity between the copied product and its template, which could be mathematically represented as the following equation.

$${\text{arg}}\,\underset{\theta }{{\text{min}}} \left(\Delta (H\left(\theta \right), T)\right)$$

where $T$ denotes the reference template; $H$ denotes the copied product; $\theta$ denotes the estimated alignment. In the equation, $H(\theta )$ represents the copied product after the estimated alignment was applied. The dissimilarity metric (*Error*), $\Delta \left(H\left(\theta \right), T\right)$, was obtained by finding the closest point in the reference template ($T$) for every point in the copied product ($H$) and calculating the average distance between $T$ and $H$ using point-to-point comparison. During the process of image registration, the CAM-VMI firstly superimposed onto the template (Fig. 1A) then fine-tuned the copied product’s size, orientation, and position and aligned it with the template with appropriate re-sizing, re-rotating, and re-positioning (Fig. 1B). Upon completion of the image registration process, two aspects that reflected the constructional problems of the copied product, namely, *Error* and *Effort*, were then obtained through pixel-by-pixel comparison. *Error*, defined as the mismatch between the best-fit image and the template (Fig. 1C), represented the constructional accuracy of the copied product (e.g., simplification, difficulty in the closure, or trembling lines) [21]. *Effor*t, $\Delta \left(H\left({\theta }_{best-fit}\right),H\left({\theta }_{initial}\right)\right)$, defined as the adjustment distance from initial position to the best-fit position (Fig. 1D). It indicated the appropriateness of the alignment of the copied products (e.g., too-large or too-small copied products, too-slanted copied products, or copied products that extended beyond the boundaries of the grid on the test sheet) [21]. Each copied product had both *Error* and *Effort* scores. For each participant, the nine *Error* and *Effort* scores were then averaged to indicate VMI ability. Higher *Error* and *Effort* scores indicated poorer VMI.

### Procedure

Participants were seated comfortably in front of a height-adjustable table in a silent laboratory and were instructed to complete the CAM-VMI and Beery-VMI. Based on the instruction of the Beery-VMI, for each form, participants were asked to see the form at the top and draw the same form in the space below. After the assessment, a certified occupational therapist (RY Chen) scored the Beery-VMI based on the manual. The CAM-VMI was scored by the computer-aided method described above.

### Data analysis

In the first tier, the group differences of the Beery-VMI, the *Error* of the CAM-VMI, and *Effort* of the CAM-VMI were examined using the independent samples *t*-test. In the second tier, a stepwise linear regression was adopted to explore potential predictors of the CAM-VMI aspects (*Error* or *Effort*) on VMI performance measured by the Beery-VMI. To further identify the significant items contributing to VMI performance, a second stepwise linear regression was subsequently applied for the aspects significantly predicting the Beery-VMI. That is, if the overall *Error* could predict the Beery-VMI, the nine *Error* scores of the CAM-VMI items could be used to build the regression model. The significance level was set at *p* ≤ 0.05. All data analyses were conducted in SPSS 20.

## Results

### Tier 1: VMI performance of school-aged children with UCP and TD children

No demographic differences were found between the UCP and TD groups (Table 1). The Beery-VMI scores of children with UCP were lower than those of TD children (95% CI [-6.50, -3.36], *t* = -6.31, *p* < 0.0001). Additionally, higher *Error* (95% CI [1.43, 6.16], *t* = 3.22, *p* = 0.002) and *Effort* (95% CI [9.18, 41.05], *t* = 3.16, *p* = 0.003) were both found in children with UCP, indicating aggravated constructional problems of their copied products.

**Table 1:** Demographic data and VMI performance of participating children

	UCP (*n* = 28)	TD (*n* = 28)	*t (df* = *54)*	Cohen’s *d*
Age in months	110.11 (24.41)	102.39 (17.11)	1.37	
Gender (Boy: Girl)	16:12	12:16	*χ*^*2*^ = 1.14	
VMI performance				
Raw score of Beery-VMI	15.11 (2.15)	20.04 (3.53)	− 6.31***	− 1.69
*Error* score of CAM-VMI	17.09 (4.42)	13.29 (4.39)	3.22**	0.86
*Effort* score of CAM-VMI	112.73 (37.03)	87.62 (19.96)	3.16**	0.84

*VMI* visual-motor integration, *UCP* unilateral cerebral palsy, *TD* typically developing, *Beery-VMI* the Beery-Buktenica Developmental Test of Visual-Motor Integration 4th edition, *CAM-VMI* the Computer-Aided Measure of Visual-Motor Integration

**p < .01, ***p < .001

### Tier 2: aspects/items of the CAM-VMI predictive of the Beery-VMI in children with UCP

The first predictive model (Table 2) demonstrated that *Effort* was not explicitly correlated with the Beery-VMI, whereas *Error* was a significant predictor of the Beery-VMI in children with UCP. The *R*^*2*^ of 0.167 indicated that 16.7% of the variance in the Beery-VMI could be explained by the overall *Error* scores (*F*(1, 26) = 5.20, *p* = 0.03). Because *Error* was the only aspect that predicted the Beery-VMI, the *Error* scores of each item were then selected as the potential factors in the subsequent predictive model (Table 3). The results indicated that only the *square* item was predictive of the Beery-VMI; the *Error* score of the copied *square* item was the only significant predictor of the Beery-VMI in children with UCP, accounting for 32.0% of the variance (*F*(1, 26) = 12.26, *p* = 0.002).

**Table 2:** CAM-VMI aspects significantly predictive of the Beery-VMI of children with UCP

Intercept *B*	Significant predictor		*F*(1, 26)	*R*^*2*^	VIF
	*Error*	*Effort*			
18.498	− 0.408*	–	5.20	0.167	1.00

Variables entered if significance of changed *F*-value ≤ .05; variables removed if significance of changed *F*-value > .10

*CAM-VMI* the Computer-Aided Measure of Visual-Motor Integration, *Beery-VMI* the Beery-Buktenica Developmental Test of Visual-Motor Integration, 4th edition, UCP, unilateral cerebral palsy

*p < .05

**Table 3:** CAM-VMI items significantly predictive of the Beery-VMI of children with UCP

Intercept *B*	Significant predictor									*F*(1, 26)	*R*^*2*^	VIF
	Vertical line	Horizontal line	Circle	Cross	Right oblique line	Square	Left oblique line	Oblique cross	Triangle			
17.249	–	–	–	–	–	0.566**	–	–	–	12.26	.320	1.00

Variables entered if significance of changed *F*-value ≤ .05; variables removed if significance of changed *F*-value > .10

*CAM-VMI* the Computer-Aided Measure of Visual-Motor Integration, *Beery-VMI* the Beery-Buktenica Developmental Test of Visual-Motor Integration, 4th edition, *UCP* unilateral cerebral palsy

**p < .005

## Discussion

A newly developed computer-aided measure of VMI was used to investigate VMI performance in school-aged children with UCP. As indicated by the CAM-VMI and Beery-VMI, children with UCP had poorer VMI performance than that of TD children. Furthermore, the predictive model demonstrated that the *Error* score obtained by the CAM-VMI reflected the VMI performance measured by the Beery-VMI. The *Error* score of the copied *square* item was the best predictor of VMI performance in children with UCP.

### Tier 1: VMI performance in school-aged children with UCP and TD children

Results of the Beery-VMI revealed impaired VMI performance in children with UCP, even when they copied the geometric shapes using the less-affected hand. This impairment was further found in both the *Error* and *Effort* aspects evaluated by the CAM-VMI. Copied products of children with UCP showed poorer constructional accuracy and required more adjustment. These characteristics might indicate that children with UCP make mistakes (e.g., scribbles, trembling lines) and alter the size, rotation, and position of the template when copying. The impaired VMI in these children might be due to the limited motor function of their less-affected hand and their impaired visual perception [22, 23]. Additionally, the children with UCP avoided the use of their non-dominant hand (i.e., more-affected hand) to stabilize the test sheet [11]. This behavior might also have reduced the quality of the copied products. The present study directly compared the VMI performance of children with UCP with that of age- and gender-matched TD children. The direct evidence confirmed the consensus that UCP adversely impacts VMI performance, supporting the need for VMI interventions for children with UCP.

The CAM-VMI, a newly-developed computer-aided measurement, was used in the present study to comprehensively assess the VMI abilities of children with UCP. The scoring method (i.e., image registration technique) used in the CAM-VMI is similar to that of the currently available computer-aided VMI assessments [13, 14]. However, the CAM-VMI extends the scoring concepts by considering not only the *Error* but also the *Effort* of constructional problems. *Error* represents the constructional accuracy of the copied product due to scribbles or trembling lines; *Effort* indicates the required adjustment of the copied product due to inappropriate size, rotation, and position. Children who perform poorly on *Effort* may not necessarily perform poorly on *Error*. For example, an oversized circle or a slanted line demonstrates high constructional accuracy (i.e., small *Error*) but requires the adjustments of resizing or rotating to match the template. In such a case, an evaluation tool which considers only constructional accuracy might overestimate the actual capability of VMI. Theoretically, shapes that are identical in size, orientation, and position to the templates may represent the best VMI performance. The CAM-VMI helps to eliminate the probability of overestimation because the aspects of *Error* and *Effort* are respectively considered. Furthermore, the all-or-nothing scoring of the Beery-VMI might be too crude an approach for sensitive determination of VMI ability, as copied products from different children but all awarded one point could still have large discrepancies in quality. Thus, the subtle differences between individuals could be investigated using the proposed CAM-VMI. The CAM-VMI appears to have potential for application to the precise and detailed analysis of further investigations of the mechanism of handwriting difficulties in children with UCP.

### Tier 2: aspects/items of the CAM-VMI predictive of the Beery-VMI in children with UCP

For children with UCP, the CAM-VMI partially reflected the VMI performance as measured by the Beery-VMI. The items selected in the CAM-VMI incorporated developmental considerations, as did those in the Beery-VMI. Therefore, it was reasonable that the more accurately the children copied the shapes in the CAM-VMI, the higher their scores on the Beery-VMI would be. However, *Error* accounted for only 16.7% of the variance of the predictive model, implying that the overall constructional accuracy of the copied products was not solely responsible for predicting Beery-VMI performance. Only nine geometric shapes were chosen for the CAM-VMI; this small item set may not have covered the entire difficulty range of the Beery-VMI items. Some geometric shapes with high difficulty should be included in the future to improve the predictability of *Error*. The CAM-VMI cannot substitute the Beery-VMI. On the other hand, *Effort* was not chosen in the predictive model of the Beery-VMI, probably due to the difference in scoring concepts. The appropriateness of alignment (e.g., size, rotation, and position) of the copied products was not emphasized in the scoring of the Beery-VMI [12], limiting the predictability of *Effort*. However, concepts similar to *Effort* have been considered in other assessments, such as the Bruininks-Oseretsky Test of Motor Proficiency, Second Edition (BOT2) and DTVP. In those assessments, similarity between the overall orientation and size of the copied product and the corresponding template are evaluated [24, 25]. The more similar a copied product is to its template, the better the VMI performance. Additionally, the concept of *Effort* is also considered in the evaluation of handwriting quality [3, 21, 26], drawing attention to the appropriateness of the alignment of the copied products when evaluating VMI.

In children with UCP, the *Error* score of the *square* item of the CAM-VMI was the most important predictor of VMI performance measured by the Beery-VMI. Copying a *square* primarily requires the ability to complete two horizontal and two vertical lines, which together compose a closed shape with four equal angles and edges. Drawing straight lines to form a perpendicular angle is challenging and requires skilled motor performance [15]. Although all the children with UCP passed this item based on the criteria of the Beery-VMI, which emphasized only the four-sidedness of the *square* or *quadrangle* [12], children with UCP often copied the *square* with undesired circular corners and curved edges. They also had difficulties in drawing four lines of equal length to close the *square*. The contribution of the *square* item to VMI performance was also reported in the factor analyses studies of the Beery-VMI [16, 17]. It appears that the ability to copy a *square* is an important indicator of children’s VMI development.

### Study limitations

This study has some limitations. First, the items of the CAM-VMI were chosen based on prewriting skills [16, 27]. Thus, they may have been too easy for older children with UCP and TD children. Modification of the test items of the CAM-VMI will be needed in the future to validate the findings. The CAM-VMI appears to be suitable for pre-school children. Pre-school children should be included in future investigations of the importance of VMI in the early stage of handwriting acquisition. Second, this study focused only on static copied products. Exploring the dynamic process (e.g., kinematics and kinetics) during shape-copying tasks might provide new insights into VMI [28]. Third, extend the current 9 shapes of CAM-VMI to encompass all 24 shapes of the Beery-VMI might enable the direct comparison between computer-aided measure and manual approach. It may help develop computer-aided Beery-VMI and improve its clinical utility. Despite the limitations, the CAM-VMI still demonstrated its potential as an evaluation tool for VMI due to the objective and precise nature of computer-aided measurement and its comprehensive scoring system. Therefore, the CAM-VMI might be helpful to elaborate the controversial findings on the relationship between VMI and handwriting skills in children with UCP.

## Conclusions

This study was a large-scale study that provided direct evidence of impaired VMI performance in school-aged children with UCP using the CAM-VMI and the Beery-VMI. Impaired VMI performance was found in the *Error* and *Effort* scores of the CAM-VMI, possibly suggesting that, compared with those of TD children, the copied products of children with UCP had poorer constructional accuracy and inappropriate size, orientation, and position. The more detailed information provided by the CAM-VMI may have greater utility than the binary all-or-nothing scoring procedure adopted by the Beery-VMI and enhance the sensitivity of VMI evaluation. Furthermore, for children with UCP, the *Error* scores of the copied shapes, particularly the *square*, best predicted the VMI performance as measured by the Beery-VMI, indicating the importance of the *square*-copying task as an indicator of VMI development in children with UCP.

## Data Availability

The datasets used and/or analyzed during the current study are available from the corresponding author on reasonable request.

## Abbreviations

Beery-VMI: Beery-Buktenica Developmental Test of Visual-Motor Integration
CAM-VMI: Computer-aided measure of visual-motor integration
TD: Typically developing
UCP: Unilateral cerebral palsy
VMI: Visual-motor integration
